# Cardiac rehabilitation in older adults: New options

**DOI:** 10.1002/clc.23296

**Published:** 2019-12-11

**Authors:** Kartik R. Kumar, Ileana L. Pina

**Affiliations:** ^1^ Division of Cardiology, Department of Internal Medicine Wayne State University Detroit Michigan

**Keywords:** cardiac rehabilitation, home based cardiac rehabilitation, older population

## Abstract

Cardiac rehabilitation (CR) is an important component in the continuum of care for patients with cardiovascular diseases, including the older population. Benefits of CR which include mortality benefit, decreased hospitalizations, increased functional capacity all extend to an older population. In Medicare beneficiaries which represent an older population, utilization of CR continues to remain low despite evidence that suggests lower hospitalization rates, Medicare costs, and improved symptoms. Given poor referral rates, enrollment rates, and completion rates, a call for new strategies has been made by all major societies. However, several barriers exist. Newer models of CR constructed to overcome these barriers are reviewed below. Some of these new strategies include alternative site CR or home‐based CR and the utilization of technology.

## 1 INTRODUCTION

Cardiac rehabilitation (CR) is an important component in the continuum of care for patients with cardiovascular diseases. CR has a class IA recommendation by the American Heart Association and American College of Cardiology^1^ for secondary prevention after a myocardial infarction, including non‐ST‐elevation myocardial infarction/unstable angina^2^ and ST‐elevation myocardial infarction, percutaneous coronary intervention, coronary artery bypass graft surgery, or in the setting of stable angina or symptomatic peripheral arterial disease. In addition, CR is also recommended after heart valve surgery, cardiac transplantation, or in the setting of chronic heart failure with reduced ejection fraction.

In the older population, CR is an incredibly powerful tool. One large study by Suaya et al examining a very large pool of Medicare beneficiaries with coronary disease, found mortality rates 21% to 34% lower in patients who utilized CR over patients who did not, which was similar to studies found in a younger population.^3^

Despite the above strong recommendations and benefit, there continues to be a significant underutilization of CR. A study by Fang et al utilized the Behavioral Risk Factor Surveillance System for 2013 and 2015 to assess use of CR and found overall use of CR was 33.7% following an AMI.^4^ In utilization data from the Get With The Guidelines‐Heart Failure registry, patients hospitalized with heart failure with reduced ejection fraction and preserved ejection fraction received 10.4% and 8.8% referrals for CR, respectively.^5^ Specifically, for Medicare beneficiaries which represent an older population, utilization of CR continues to remain low^6^, ^7^ despite evidence that suggests lower hospitalization rates, Medicare costs, and improved symptoms. CR has been shown to have significant aggregate mortality and morbidity benefits in addition to improvements in exercise capacity, blood pressure, lipids, inflammation, and psychosocial stress.^8^, ^9^, ^10^ In addition, quality of life scores are also significantly improved in an older population >70 years of age with coronary heart disease who participate in CR.^11^ Similarly, older patients with coronary heart disease and depression who enroll in CR have significant reductions in the prevalence and severity of this psychiatric disorder.^12^

The American College of Cardiology and American Heart Association have recently published a comprehensive list of measures to be used for eligible patients which includes six performance measures and three new quality measures. This document also acknowledges that alternative models of CR delivery are both feasible and potentially helpful to expand the reach of CR services.^13^

Cardiac rehabilitation has primarily been offered as a center‐based protocol and this model has been well established to reduce hospital readmissions, secondary events, and cardiovascular mortality in patients with coronary heart diseases.^14^, ^15^ Benefits of CBCR has been limited by underuse among eligible patients. An effort to increase the older populations participation in CR has been an especially challenging goal. Reasons for poor participation in this population may be totally unrelated to medical issues but rather to socioeconomic difficulties including transportation to CR centers. Ades et al attempted to explore predictors of CR participation in older patients with coronary artery disease. The study included 226 eligible patients with a participation rate of 21% (47/226) and found that patients who participated in CR were younger (68 ± 5 vs 71 ± 6), lived closer to the rehabilitation program, and were more likely to own and drive a car, as compared to the nonparticipants. Other important predictors of participation included education of participants. Multiple comorbidities and psychosocial factors such as denial of severity of disease and depression were also predictors of nonparticipation, all of which are more evident in the older population. However, the most powerful predictor of participation in this study was the strength of the primary physicians recommendation.^16^

Current CR programs may be center‐based CR, including inpatient, outpatient‐hospital or facility based, or home‐based cardiac rehabilitation (HBCR) each rehabilitation model has its benefits and limitations. However, given the trend for shorter hospital stays, decreased priority of inpatient programs, and an effort to increase overall participation in CR with more convenience, an increased focus on outpatient rehabilitation programs has emerged. HBCR vs CBCR also differ in standard and quality but important benefits of the former include ease of transportation and logistics which is a key issue with the older population. Insurance coverage is again, another important consideration when determining the appropriate program for the patient.

Cardiac rehabilitation has quickly developed into more than just a physical exercise routine. Rather, it has become a comprehensive multifaceted program, of which one part is physical exercise. Smoking cessation, medication adherence, healthy dietary habits, and stress management have all been found to be important in current CR programs (Table 1). Given the continued poor referral rates, enrollment rates, and completion rates, a call for new strategies has been made by all major societies.^17^ To achieve this comprehensive approach, alternative sites for CR, technology, and novel therapies are all being actively investigated for utilization, some of which will be reviewed below.

**Table 1:** Cardiac rehabilitation has transformed beyond simple physical activity

Cardiac rehabilitation targets for improvement
Aerobic exercise
*Smoking cessation*
*Dietary management*
*Medication adherence*
Stress management
Physical activity

## 2 HOME‐BASED CARDIAC REHABILITATION

Home‐based cardiac rehabilitation was implemented to increase the participation in CR by theoretically overcoming common barriers such as geographic, logistical which may include transportation, scheduling, center capacity among others, and other related barriers. HBCR has always been advocated for when patients are unable to attend CBCR, however, a standalone program is still developing, albeit at a rapid pace. Multiple updated systematic reviews of current literature in the Cochrane collaborative reviews of CR have concluded that there is low‐ to moderate‐strength evidence that HBCR and CBCR have similar effects on quality of life and cost among patients with a recent MI or coronary revascularization.^18^, ^19^

Specific to the population ≥65 years of age, HBCR was an important focus of research as participation in CR is particularly low in this age group. The home‐based approach to support the elderly population with cardiac disease was seen as early as 2005 by Sinclar et al who utilized a home visiting nurse to stress the basic tenets of CR, knowledge of treatment regimens, risk factor reduction, exercise and stress management, smoking cessation, and diet. The patients with home visiting nurse had improvement in self‐reported quality of life questionnaire as well as reduced rehospitalizations.^20^ In a randomized control trial by Oerkild et al, with a mean age of 74.4, HBCR was shown to be as effective as CBCR in elderly with coronary heart disease with no differences in improvements to peak VO_2_, 6‐minute walk test, blood pressure benefits, decreased cholesterol, and health‐related quality of life.^21^ In another follow‐up study by Oerkild, in which a similarly aged population who declined CBCR and were offered HBCR and compared to patients who declined both CBCR and HBCR. This study showed similar improvements as the former study and noted that HBCR was a feasible option in elderly with high levels of comorbidity and low levels of exercise, often reasons for noneligibility for CBCR.^22^

Another interesting advantage of HBCR would be the potential for a more comprehensive educational outreach for the >5000 waking hours patients spend each year independent of medical providers. This expanded contact with the patient could allow for more effective behavioral change strategies in regards to tobacco cessation, dieting, and other factors which cannot be wholly addressed in the limited 3 to 4 hours per week sessions done in most CBCR programs.^23^ Utilizing newer technologies such as automated pill dispensers, which have been shown to improve adherence,^24^ or mentorship and support from other patient peers who have previously faced similar circumstances with good outcomes are targets for the significant amount of time the patient is not directly supervised by medical personnel. Another important barrier to CR which is potentially overcome by HBCR is patient enrollment and completion. A recent study which gave CR eligible patients the choice between CBCR or HBCR, nearly half chose a home‐based approach.^25^ Adherence to HBCR has also been compared to CBCR and while no definitive data exists due to inconsistent reporting of adherence, a Cochrane review by Anderson et al found slightly higher level of adherence with HBCR than CBCR as well as completion rates of the prescribed CR in the HBCR participant groups compared with CBCR.^18^ In addition to the above benefits, it has been suggested that lifestyle changes that occur during CBCR will decrease at the conclusion of the CBCR intervention. In contrast, HBCR programs which involve a higher level of self‐monitoring and disease management by the patient, may lead to a more durable and potentially lifelong lifestyle intervention. In fact, one study evaluating exercise tolerance by a metric of total work capacity (TWC) found that while both CBCR and HBCR both increased TWC, patients in the CBCR tended to regress toward baseline toward the end of the 12‐month program, whereas TWC was maintained in the HBCR.^26^

A recent scientific statement from the American Association of Cardiovascular and Pulmonary Rehabilitation, AHA, and ACC regarding HBCR has advocated for HBCR as an alternative option in selected clinically stable low to moderate risk patients who may otherwise not be able to attend CBCR.^27^

While the advantages of HBCR have been described above, several limitations do exist (Table 2). Concern regarding HBCR focused initially on the safety of initiating an exercise regimen in an environment without supervision. Compared to CBCR, studies in low to moderate risk patients appear to have similar cardiovascular event rates. Even in an older population, HBCR appears to be just as safe as CBCR.^26^ Of note, significant cardiovascular events with CBCR was low to begin with so the power of these studies with HBCR is likely low.

**Table 2:** Potential advantages and disadvantages of home‐based cardiac rehabilitation vs center‐based cardiac rehabilitation

Advantages	Disadvantages
Improvement in time to enrollment	Lack of reimbursement by all insurers
Individually tailored	Less intensive exercise training
Expanded capacity and access	Less social support
Patient friendly scheduling and flexibility	Heavier patient self‐reliance
Minimal travel/transportation limitation	Lack of a standard HBCR protocol
Greater privacy	Less face‐to‐face monitoring and communication
Integrates with regular home routine	Safety concerns in higher risk patients

*Note*: Adapted from Thomas et al home‐based cardiac rehabilitation.

Abbreviation: HBCR, home‐based cardiac rehabilitation.

Other limitations include continued participation and engagement in a HBCR model. As evidenced in the HF‐ACTION trial, adherence to the home‐based portion of the trial was low, in spite of heart failure patients having been given exercise equipment at home to use and to keep in addition to phone calls, and diaries, among other reminders.^28^

## 3 HYBRID CARDIAC REHABILITATION—CENTER BASED AND HOME BASED

The HF‐ACTION trial which included patients with heart failure with reduced ejection fraction, studied a hybrid method of CR. Patients randomized to exercise training participated in 36 supervised sessions with transition to a hybrid (HBCR + CBCR) model after their 18th session and fully HBCR after completion of 36th session. The study concluded that exercise training in a hybrid model was safe with systolic heart failure. In addition a nonsignificant reduction in a composite end point of all‐cause mortality and all‐cause hospitalization was identified, however, after adjusting for certain baseline characteristics which were highly prognostic for the primary end point (duration of exercise, left ventricular ejection fraction, Beck Depression Inventory II score, and history of atrial fibrillation) there was a significant reduction in the primary end point with exercise training. Of note, the study population group was younger with a mean age of 59, however, a significant portion were >60 years old.^28^ However, by one study, participation in a HBCR program was found to be inversely associated with age, however, age was not associated with graduation or completion from the program by one study.^29^

A recent clinical trial which is currently enrolling participants is seeking to evaluate a hybrid CR model, specifically in an older population, and is called the Modified Application of Cardiac Rehabilitation for Older Adults (MACRO). The trial aims to provide personalized engagement, deprescribing, and focus on facilitation of enrollment in CR to a site that best suits each participant which may include a supervised regimen, a home based or self‐monitored program, or both.^30^

## 4 HBCR WITH TELEPHONIC MONITORING

Telephonic monitoring with HBCR has been suggested to be beneficial for many years. An early study with a small population using telephonic exercise monitoring found that it could be a useful alternative to CBCR.^31^ This includes a study by Wakefield et.al, conducted in a Veteran population with a mean age of 63.7 ± 8.2 utilizing a remote, HBCR as well as periodic telephone calls. The study concluded that this model would not only bring CR services closer to patients, but noted participants were highly satisfied and had higher completion rates.^32^

Larger studies utilizing telephonic monitoring and mail contact with patients have shown to improve several coronary risk factors such as lipid profiles, activity, and smoking cessation, after acute myocardial infarction and the implementation of this could be useful for comprehensive CR.^33^, ^34^ In addition, in a study specifically following a CR program with telephone follow‐ups found that the intervention resulted in a significantly improved Framingham score, total cholesterol, low‐density lipoprotein cholesterol, and systolic blood pressure when compared to patients who completed CR without any follow up.^35^

A study published in 2010 evaluating a home‐based telephonic monitoring CR program, which included a mobile transmitted ECG and telephonic contact for psychological support, training sessions, and baseline assessment, vs a standard CR protocol at a facility or hospital found that 100% of patients in the home‐based group completed the 8‐week course while 20% of the standard group had dropped out for several of the typical reasons including financial, transportation, personal life conflicts. Efficacy of the programs was also found to be equal.^36^Post discharge heart failure disease‐management programs are available at many hospitals and rely heavily on telephonic monitoring. In a study by Berg et al in a community population with a mean age of 76.2 evaluating this management program which includes telephonic monitoring by a registered nurse at scheduled times, education sessions, symptom advice line, work books, individualized assessments, medication compliance reminders, and vaccination reminders, as well as physician alerts found reductions in hospitalization as well as reductions in utilization of medical services which resulted in lower costs of care.^37^

## 5 DIGITAL, SMARTPHONE, AND INTERNET‐BASED CARDIAC REHABILITATION (eCR)

Cardiac rehabilitation has progressed from a simple physical component of increasing exercise tolerance to a more comprehensive approach including, diet, medication compliance, smoking cessation, and psychosocial support. Accordingly, newer interventions utilizing common place technology and outside of standard center‐based physical therapy is vital for the success of future CR pursuits. Remote digital CR is a new and exciting form of delivering CR to those who face the typical barriers to CR.

With the above goals in mind, a small feasibility study was performed in 2014 involving 26 patients, 33% of which were >65 years of age, which noted integrating a mobile care delivery platform into a CR program was feasible, safe, and agreeable to patients and clinicians.^38^

A recent systematic review of mobile health applications identified 12 studies focusing on CR. Eleven different smart phone applications were identified and intended to facilitate CR in the home by improving medication compliance, exercise, appointment compliance, symptom monitoring, and physical activity monitoring via GPS. Behavior modification and messaging with healthcare providers was also noted in some applications.^39^

This innovative approach to increasing utilization and compliance with CR through technology is applicable to all age groups. A recently published survey of 200 patients being discharged following percutaneous coronary intervention, cardiac surgery, or acute coronary syndrome found that a remote digital CR program would be acceptable to most cardiac patients, including the older population.^40^

A randomized controlled trial also showed a significant benefit of digital health intervention when added to standard CR. The study utilized smartphone and online‐based CR platforms and asked patients regarding daily exercise and dietary routines. The digital health intervention in combination with CR had more reduction in weight when compared to CR alone. There was also a nonstatistical reduction in cardiovascular related visits to the emergency department.^41^

As a standalone eCR, in a randomized control trial, a smartphone‐based home care model was compared to a CBCR program with primary outcomes such as CR uptake, adherence and completion of the CR program. Significantly more participants completed the eCR program (80%) than the CBCR (47%). In the study, eCR was as effective as HBCR in improving health outcomes including functional capacity, dietary compliance and decreased depression. Participants in the eCR group were 54.9 ± 9.6 years of age.^42^

Incorporating digital, smartphone, and internet‐based strategies for CR in the elderly is limited by a decreased adoption and use of these devices and technologies. A study of smartphone ownership by the Pew Research Center reveal that only 53% of adults ≥65 years of age own a smartphone compared to 96% of 18‐ to 29‐year‐old and 79% of 50 to 64 years of age.^43^ Despite this, by one small study of adults aged 65 to 76 years old who were relatively naïve to technology, especially tablets, the participants were eager to adopt the new technology, however, were apprehensive about lack of clarity in instructions and support. Understanding this perception and implementing eCR with this in mind may find increased support in this age group.^44^

## 6 OTHER NOVEL METHODS OF CR

Newer methods of augmenting the benefit achieved from time spent in CR programs have become a focus of study. A recent randomized control trial evaluating standard continuous aerobic training to super‐circuit training, defined as aerobic interval exercise combined with alternating sets of resistance training, in postmyocardial infarction patients, concluded that this form of exercise yielded greater reductions in *E*/*e*′ and an increase in ejection fraction. In addition, increased metabolic equivalents and physical component of quality of life were also greater in the super‐circuit training group.^45^

Other methods of exercise have also been postulated to improve outcomes and also assist patients in increasing participation in CR who would otherwise have said no due to the misconception of excessive vigor and risk associated with it. A study by Salmoirago‐Blotcher et al evaluated a 6 month tai chi program vs a 3 month less rigorous program, in patients who declined CR and noted safety, improved physical activity, weight, and quality of life in the 6 month group.^46^

Art in its various forms has also been evaluated as a possible component of cardiovascular rehabilitation. Music, sculpture, and paintings, stimulate neural activity which may play a role in regulating the sympathetic and parasympathetic tones to the cardiovascular system. Music, appreciation of art of in a Museum setting, and even painting or coloring have been utilized for a wide spectrum of purposes including relaxation, stress care, repression of loneliness, and active life motivation which may translate to lowering blood pressure and control of higher heart rates.^47^ Music has been studied in individuals with coronary heart disease and has been utilized to reduce anxiety and distress as well as improve physiological functioning in medical patients. A Cochrane Database Systematic Review by Bradt et al indicates that music may have a beneficial effect on anxiety, especially with those with myocardial infarction. It also noted a likely beneficial effect on systolic blood pressure, heart rate, respiratory rate, quality of sleep, and pain in patients with coronary heart disease, however, clinical significance is unclear and further studies are needed.^48^

### 6.1 CR for newer and potential indications

The US Centers for Medicare and Medicaid Services added peripheral artery disease (PAD) as an indication for CR in the form of supervised exercise therapy on May 25, 2017. The prevalence of PAD in the elderly is noted to be approximately 18% by one study in patients aged >55.^49^ In addition, the prevalence of PAD increases with age with approximately 7% of patients 70 to 74 years of age having symptomatic PAD with intermittent claudication.^50^ Exercise training is a first line recommendation for symptomatic PAD^51^ and the benefits extend to beyond improvement in intermittent claudication. Exercise training in patients with PAD who completed a 12‐week supervised exercise program showed a higher cardiovascular death‐free rate higher in patients who completed the program vs those who did not.^52^

CR for heart failure is reimbursed for reduced ejection fraction (HFrEF), however, is not for patients with preserved ejection fraction (HFpEF) per the Centers of Medicare and Medicaid. Importantly, patients with HFpEF are older and thus this population is disproportionately excluded from the benefit of CR. However, recent evidence is suggesting there is a benefit in these patients, particularly the elderly. In a recent study by Pandey et al evaluating the response to endurance exercise training in older patients (mean age 69.6 ± 5.5) with HFrEF compared to patients with HFpEF the investigators found that improvement in peak VO_2_ was considerably better in patients with HFpEF. In addition, there was a higher proportion of clinically meaningful improvement.^53^

## 7 INCREASING CR UTILIZATION

It is important to note that there are a known set of geriatric variables that must be contended with when pursuing CR in this fast‐expanding population. Frailty and multimorbidity, which could include multiple coexisting cardiovascular conditions in addition to noncardiovascular, polypharmacy, deleterious effects of medical care including posthospital deconditioning, delirium and disability, sarcopenia, and declining cognition.

## 8 FRAILTY

A systematic review of studies including 54 250 elderly patients with cardiovascular disease found the prevalence of frailty to be 50% to 54%.^54^ Patients with frailty were generally excluded from rehabilitation studies as they have lower than required baseline exercise capacities or sarcopenia. Also, a general attitude of frail patients being too unfit for rehabilitation has become prevalent.^55^ Frailty must be integrated into the concept of CR and an assessment should be made for frailty including physical performance tests and should be managed accordingly to maximize the benefits of CR.^56^ An ongoing study seeks to evaluate a combination of both HBCR as well as leveraging technology in the form of a necklace‐worn sensory to evaluate physical activity stimulation and progressively increase activity as adherence improves.^57^, ^58^, ^59^

## 9 FINANCIAL INCENTIVES

Another important limitation for achieving meaningful CR in the older population is the fact that many HBCR programs are not reimbursed by many insurers. Financial incentives to all involved parties are an ongoing topic of research to increase CR utilization and completion rates.

On December 20, 2016, the Centers for Medicare and Medicaid announced a financial incentive for institutes that provide CR. An initial $25 dollars for the first 11 sessions for CR following an acute myocardial infarction or coronary artery bypass graft would be paid for by Medicare. After the completion and payment of these 11 sessions, $175 per service would be paid up to a total of 36 sessions. However, this incentive model was shortly canceled less than a year later in November of 2017, before its proposal to go live in 2018.

Alternatively, financial incentives to the patient to encourage enrollment, participation, and completion have also been studied. A small study in 2016 put CR eligible patients with Medicaid insurance coverage on an escalating incentive schedule contingent on attendance of CR compared to a usual care group of Medicaid patients eligible for CR. Of the small study group, all participants completed at least one session of CR compared to 25% in the control group. Adherence as higher in the intervention group with an average of 31.1 sessions completed compared to 13.6 in the control group. Completion of all 36 recommended sessions was 80% in the intervention group and 8% in the control group.^60^ A 2019 randomized clinical trial by the same author Gaalema et al recruited a larger number of patients and found similar improvements in CR participation, when offered a financial incentive, among lower socioeconomic status patients following a cardiac event.^61^

## 10 CONCLUSION

Cardiac rehabilitation was primarily an exercise training program for a younger population with cardiovascular disease. However, CR has evolved into a comprehensive lifestyle program including physical activity, education, diet, risk reduction, and adherence to prescribed medical therapies. In addition, the eligible population has significantly aged. A comprehensive evaluation of all comorbidities, frailty, social, and financial factors must now all be considered in order to tailor CR to this population.

Novel approaches to overcome the common barriers are the focus of current CR research and this review. HBCR with or without telephonic monitoring need to be developed in order to increase capacity, participation, completion, and extended benefits of self‐reliance. The utilization of technology, including smart phone applications and the internet, which have become common place, is an expanding area of research and is increasingly accepted by the older population. In addition, financial incentives to all involved parties including patients, institutes, and healthcare workers is also a promising approach to overcome referral, enrollment, and completion barriers. The transformation of CR from a relatively simplified exercise regimen to a more comprehensive lifestyle and behavioral rehabilitation, demands that more tools will be required to achieve this goal. Further research to assist in tailoring an appropriate CR regimen utilizing many of the mentioned tools for the older population will be necessary to provide a not only comprehensive but also cost‐effective program. Finally, an expansion of CMS coverage for HFpEF is disproportionately affecting the older population and must be addressed.

## CONFLICT OF INTEREST

The authors declare no potential conflict of interests.
